# Assessment of Antimicrobial Activity of Lycopene, Vitamin E, and Lycopene-Vitamin E Combination Against Staphylococcus aureus, Streptococcus mutans, Enterococcus faecalis, and Candida albicans: An In Vitro Study

**DOI:** 10.7759/cureus.42419

**Published:** 2023-07-25

**Authors:** V Divyadharsini, TN Uma Maheswari, Rajeshkumar S

**Affiliations:** 1 Oral Medicine and Radiology, Saveetha Dental College and Hospitals, Chennai, IND; 2 Pharmacology, Saveetha Dental College and Hospitals, Chennai, IND

**Keywords:** zone of inhibition, tomato, carotenoids, alpha tocopherol, antibacterial

## Abstract

Background

Lycopene is a naturally occurring compound classified as a carotenoid, a group of pigments responsible for the vibrant colors observed in many fruits and vegetables. It is most commonly associated with red-colored fruits and vegetables, such as tomatoes, watermelon, pink grapefruit, and papaya. Vitamin E encompasses a group of chemical compounds that share a structural relationship with alpha-tocopherol and are essential for the proper functioning of the human body. It is a fat-soluble vitamin and is known for its antioxidant properties. The aim of this study is to evaluate the antimicrobial activity of lycopene extract, vitamin E extract, and their combination against oral pathogens for their potential application in the treatment of oral diseases.

Materials and methods

The potential antimicrobial effects of extracts derived from lycopene, vitamin E, and their combination were evaluated against oral commensals like *Staphylococcus aureus*, *Streptococcus mutans*, *Enterococcus faecalis*, and *Candida albicans*. Three concentrations (25 μl, 50 μl, and 100 μl) of the extract were tested. Mueller-Hinton agar (MHA) and Rose Bengal agar (RBA) bases were utilized to determine the zone of inhibition. And the experiments were repeated in triplicate for each group.

Results

The identification and assessment of the antimicrobial activity of lycopene extract, vitamin E extract, and their combination revealed the greatest efficacy at the highest concentration (100 μl) against all tested microbial strains. Notably, *C. albicans* exhibited the highest susceptibility compared to the other strains. Vitamin E had the least antimicrobial effect and combination had the highest antimicrobial effect.

Conclusion

The results of our study demonstrated substantial antimicrobial activity of lycopene and vitamin E. These findings suggest that lycopene and vitamin E can be harnessed in the development of diverse drug formulations for the treatment of oral diseases.

## Introduction

Lycopene is a naturally occurring compound classified as a carotenoid and is predominantly found in red-colored fruits and vegetables such as tomatoes, pink grapefruit, watermelon, papaya, and guava [1]. It is responsible for the red and orange pigmentation observed in these foods [2]. Unlike other vitamins and minerals, the human body cannot synthesize lycopene, and should be supplemented through the daily diet. It is worth noting that lycopene bioavailability may be reduced with age and certain pathological conditions, such as cardiovascular diseases [3]. As a powerful antioxidant, lycopene has attracted significant attention due to its potential health benefits. It is known for its ability to scavenge free radicals and combat oxidative stress, which can contribute to various chronic diseases, including cancer, cardiovascular disease, and age-related macular degeneration [4].

Vitamin E encompasses a group of chemical compounds that share a structural relationship with alpha-tocopherol and are essential for the proper functioning of the human body. It is a fat-soluble vitamin and is known for its antioxidant properties [5]. Alpha-tocopherol is the most biologically active form of vitamin E and is commonly found in dietary sources. It plays a crucial role in protecting cells from oxidative damage caused by free radicals, which can lead to various health problems such as oxidation of low-density lipoprotein (LDL) cholesterol, macular degeneration of the eye, neurodegenerative disorders, and acceleration of the aging process. Vitamin E also helps support the immune system and aids in the proper functioning of blood vessels. Vitamin E is considered essential for human health, and deficiencies in this vitamin can lead to various health problems, although they are relatively rare [6].

Lycopene and vitamin E combination was first tried in atherosclerosis patients. It had effective antioxidant activity against LDL oxidation and attenuated atherosclerosis. In a study by Liu et al., they compared different combinations of lycopene and concluded that lycopene with vitamin E, lycopene with vitamin C, and lycopene with beta-carotene, showed significant synergistic effects [7]. Lycopene and vitamin E, when given as a combination therapy, decreases the growth of PC-346C in prostate cancer cells [8]. 

We could not find any studies assessing the antimicrobial property of a combination of lycopene and vitamin E in oral pathogens. The rise of drug resistance has become a widespread issue, particularly concerning oral microflora, as the demand for new and highly efficient drugs continues to escalate. We thereby undertook this study to evaluate the antimicrobial effects of lycopene extract, vitamin E extract, and their combined formulation.

## Materials and methods

Lycopene preparation

Lycopene extract powder (L9879), with a purity concentration of 90%, was obtained from Sigma-Aldrich. A digital weighing machine from Shimadzu (Shimadzu Corp., Kyoto, Japan) was used to measure 2 grams of lycopene, which was then dissolved in 100 ml of deionized water. The compound was thoroughly mixed and subsequently boiled at 90°C in a Labquest by Borosil heating mantle until the aqueous solution became concentrated. The concentrated mixture underwent filtration, first by muslin cloth, and then by Whatman filter paper, and the resulting filtrate was further heated until its volume was reduced to 2 ml.

Lycopene and vitamin E combination

Vitamin E (PHR1031), with a purity concentration of 95.5%, was obtained from Sigma-Aldrich. To prepare the combination, 2 ml of concentrated lycopene compound was carefully mixed with 2 g of vitamin E. The compounds were mixed using a vortex from Starlab to ensure the complete dissolution of all the individual components. Once fully dissolved, the combination was ready for further experimental investigations.

Antimicrobial property

The antibacterial effects of lycopene extract, vitamin E extract, and their combination were assessed against oral commensals like *Staphylococcus aureus*, *Streptococcus mutans*, *Enterococcus faecalis*, and* Candida albicans*. The organisms *S. aureus* and *S. mutans* were isolated from saliva samples using special media (Mutans-Sanguis agar) and maintained in tryptone soya agar at 4°C in the Department of Microbiology, Saveetha Dental College and Hospitals, Chennai, India. *E. faecalis* and *C. albicans* were purchased from the Microbial Type Culture Collection and Gene Bank (MTCC), Chandigarh, India. The cultures were cultivated in nutrient broth and maintained in agar slants. To determine the zone of inhibition, Mueller-Hinton agar and Rose Bengal agar bases were employed in this antimicrobial activity study. The measurement of the zone of inhibition was carried out by using a physical ruler like a meter scale. The scale was placed above the Petri dish and the value of the diameter was read using the human eye. Each experiment was repeated three times for each group. All the chemicals, media, and analytical reagents used in this present work were purchased from Hi-Media Laboratories Pvt. Ltd (Mumbai, India).

Mueller-Hinton agar (MHA) 

To prepare the MHA plates, sterilization was performed for 45 minutes. The plates were then filled with the media and allowed to solidify. A fresh culture of *S. aureus*, *S. mutans*, and *E. faecalis* was grown and uniformly spread using sterile cotton swabs on Petri plates containing MHA medium. A well cutter was used to create wells on the agar plates, and lycopene, vitamin E, and their combination were loaded into the wells at different concentrations (25μl, 50μl, and 100μl). The plates were incubated at 37°C for 24 hours and the zone of inhibition was measured. 

Rose Bengal agar (RBA) 

To culture *C. albicans*, RBA, a selective medium for detecting and enumerating yeasts and molds, was utilized. For this study, a neutral pH medium supplemented with antibiotics was chosen to promote fungal growth. This selection offers an advantage over acidified media, which may inhibit fungal growth, potentially limiting the size of mold colonies. *C. albicans* was isolated and incubated in RBA supplemented with chloramphenicol at 37°C for 24 hours. After the incubation period, the zone of inhibition was measured in each group.

Statistical analysis

SPSS software (Version 9.05, Chicago, IL, U.S.A) was used to perform the statistical analysis. Repeated measures ANOVA with post hoc Tukey-Kramer test was used to compare the zone of inhibition for multiple independent groups. Variations among the sample resources were evaluated using Duncan's multiple comparison test, with a significance level set at p<0.05.

## Results

**Figure 1:**
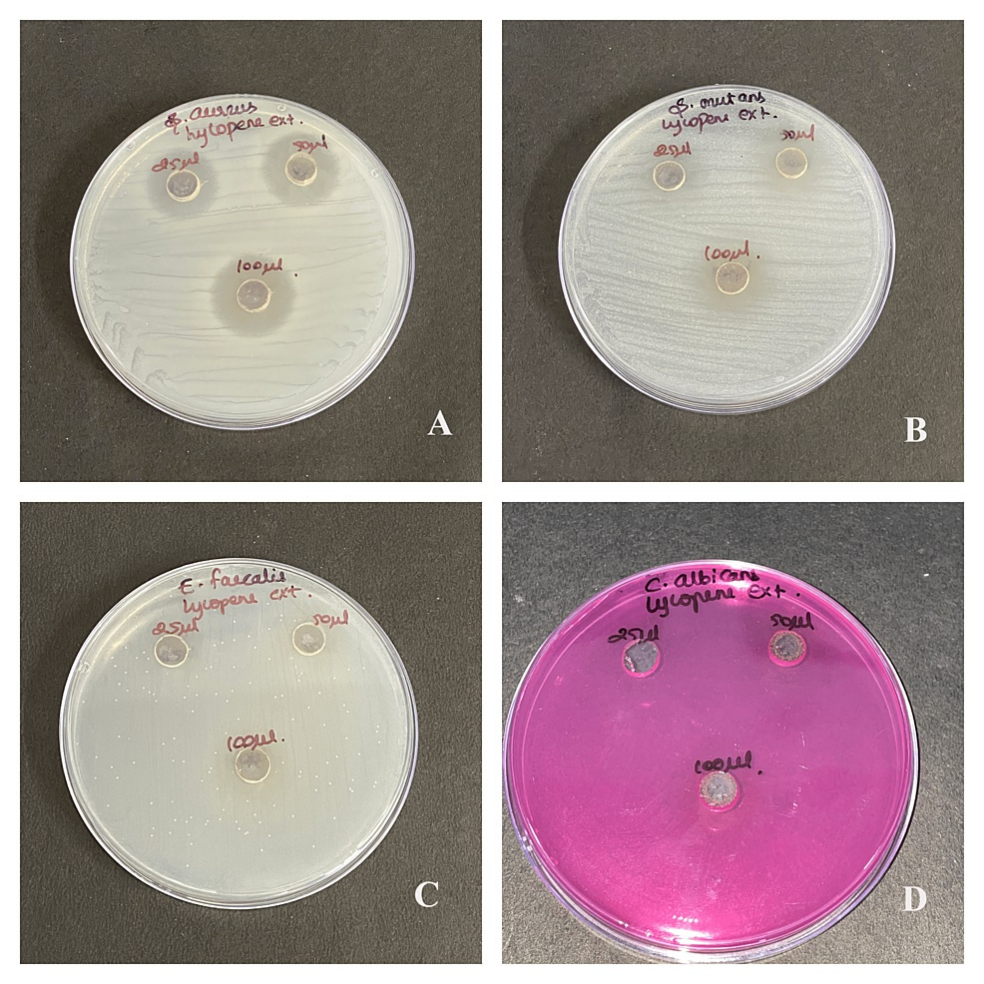
Antimicrobial activity of lycopene extract Antimicrobial activity to lycopene extract against (A) *Staphylococcus aureus*, (B) *Streptococcus mutans*, (C) *Enterococcus faecalis*, and (D) *Candida albicans* at three concentrations of 25 μl, 50 μl, and 100 μl

**Figure 2:**
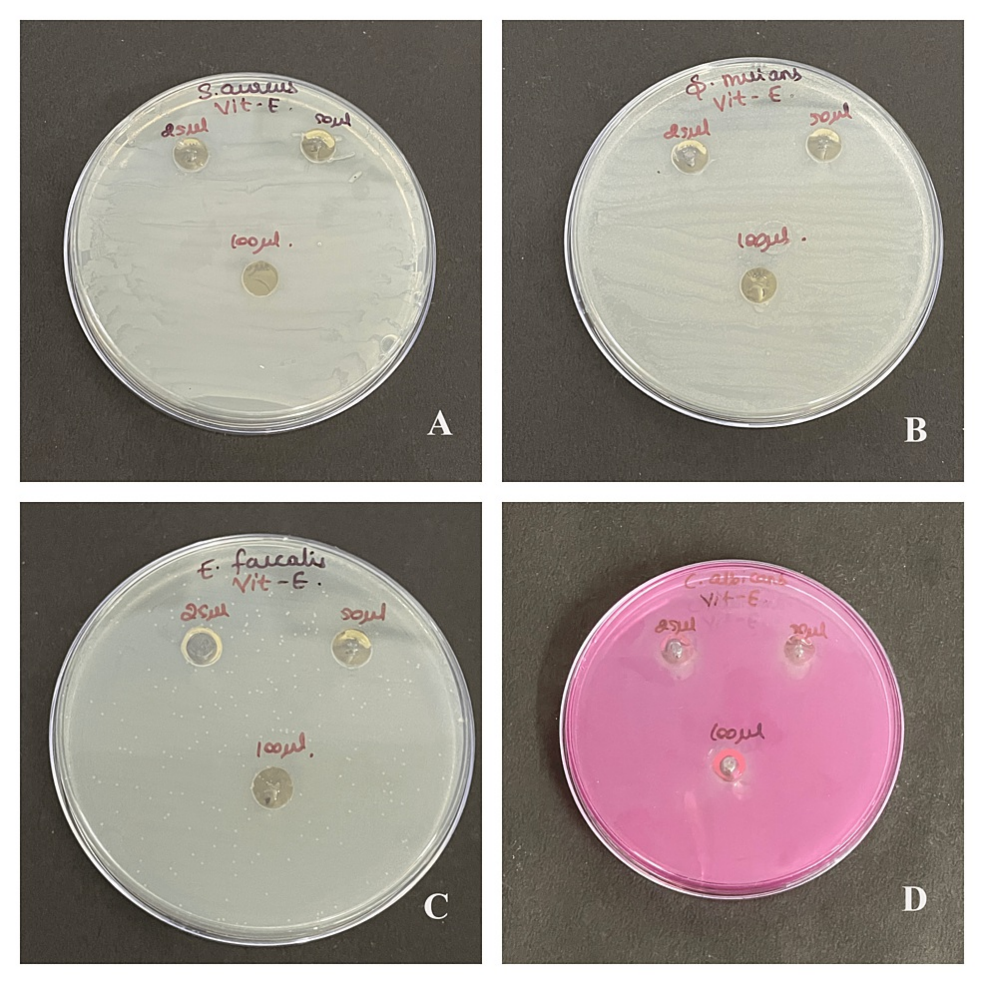
Antimicrobial activity of vitamin E extract Antimicrobial activity to vitamin E extract against (A) *Staphylococcus aureus*, (B) *Streptococcus mutans*, (C) *Enterococcus faecalis*, and (D) *Candida albicans* at three concentrations of 25 μl, 50 μl, and 100 μl

**Figure 3:**
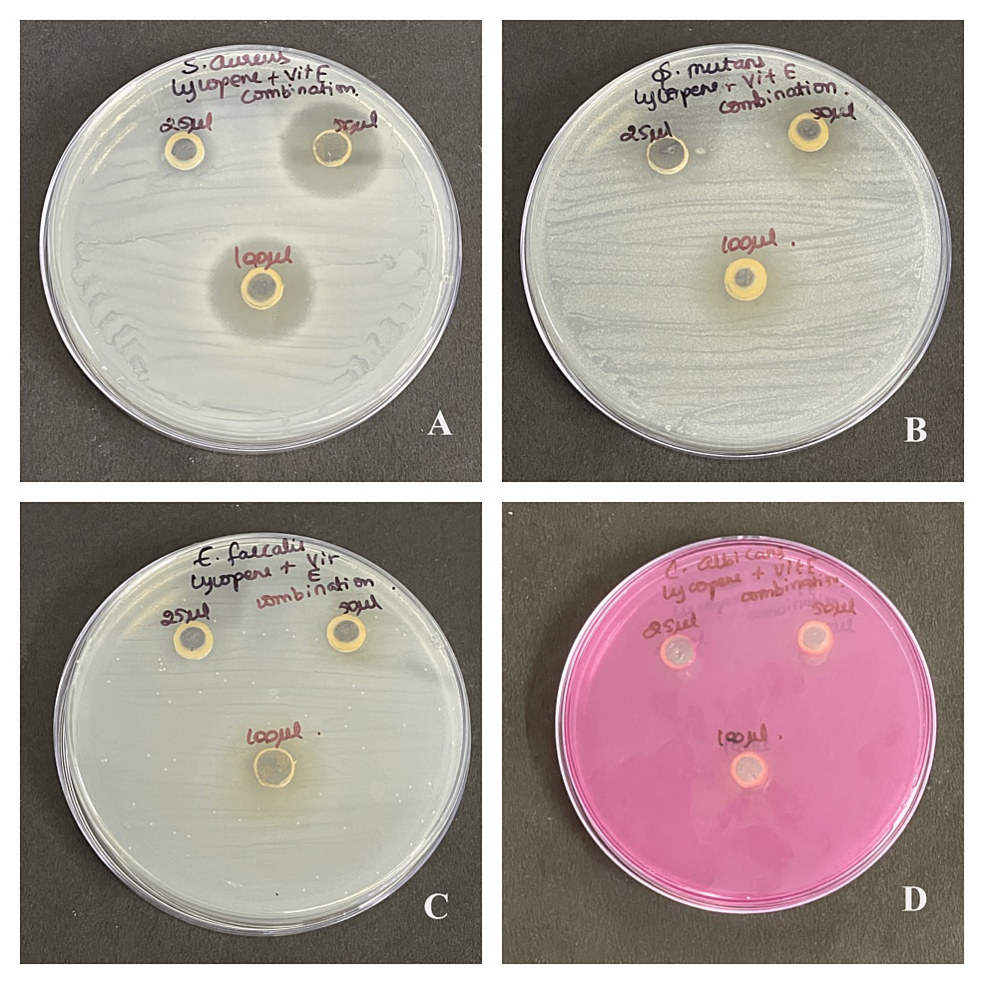
Antimicrobial activity of lycopene and vitamin E combination Antimicrobial activity to lycopene and vitamin E combination against (A) *Staphylococcus aureus*, (B) *Streptococcus mutans*, (C) *Enterococcus faecalis*, and (D) *Candida albicans* at three concentrations of 25 μl, 50 μl, and 100 μl

The antimicrobial susceptibility test showed that oral commensals like *S. aureus*, *S. mutans*, *E. faecalis,* and *C. albicans* were susceptible to lycopene extract, vitamin E extract, and lycopene-vitamin E combination by having a clear zone of inhibition, as shown in Figure 1, Figure 2, and Figure 3.

Maximum antibacterial activity was shown in the highest concentration (100 μl) in each type of microbes that were used. Moreover, *C. albicans* was the most affected than the rest. Better antimicrobial properties were observed as the zone of inhibition increased with the concentration of lycopene. There is a significant difference in the antimicrobial property of individual compounds and their combination for *S. aureus* (p=0.042), *S. mutans* (p=0.038), *E. faecalis* (p=0.022), and *C. albicans* (p=0.011) as seen in Table 1. There is a significant difference in the zone of inhibition of *S. aureus* between lycopene with vitamin E and combination, and vitamin E with combination. For *S. mutans* there is a significant difference between vitamin E and combination. For *E. faecalis* there is a significant difference between lycopene with vitamin E, their combination, and vit-E with the combination. For *C. albicans* there is a significant difference between lycopene with vitamin E, their combination, and vitamin E with the combination.

**Table 1:** Zone of inhibition at various concentrations of lycopene, vitamin E, and their combinations SD: Standard deviation

Organism	Concentration	Mean ± SD			P value
		Lycopene	Vitamin E	Combination	
S. aureus	25 μl	19.00 ± 2.000	9.33 ± 0.577	15.0 ± 1.000	0.042
	50 μl	19.67 ± 1.528	9.00 ± 0.577	21.67 ± 0.577	
	100 μl	23.67 ± 1.528	9.33 ± 0.577	22.67 ± 0.577	
S. mutans	25 μl	11.33 ± 1.528	9.33 ± 0.577	12.67 ± 1.155	0.038
	50 μl	12.67 ± 0.577	10.33 ± 1.528	15.67 ± 1.528	
	100 μl	14.67 ± 1.155	12.67 ± 3.215	16.67 ± 1.528	
E. fecalis	25 μl	11.67 ± 0.577	9.33 ± 0.577	12.00 ± 1.00	0.022
	50 μl	15.00 ± 1.000	10.00 ± 1.000	16.33 ± 0.577	
	100 μl	18.33 ± 2.082	10.67 ± 2.082	19.00 ± 1.00	
C. albicans	25 μl	20.00 ± 2.000	10.00 ± 1.000	21.33 ± 1.528	0.011
	50 μl	28.33 ± 1.528	12.00 ± 2.000	25.33 ± 0.577	
	100 μl	34.00 ± 1.000	13.67 ± 1.155	28.67 ± 1.155	

## Discussion

Lycopene offers advantageous effects in addressing specific diseases of the oral cavity, such as oral cancer and precancerous lesions. It is important to note that lycopene does not exhibit pro-vitamin A activity [9]. The role of antioxidants in combating bacterial inflammation is increasingly being acknowledged, and they have shown synergistic effects with existing antibacterials against resistant strains of bacteria [10]. Lycopene, known for its potent antioxidant properties, acts as a scavenger for reactive oxygen species (ROS), effectively preventing lipid peroxidation and DNA damage [11]. However, it has been observed that at higher incubation concentrations (>2 μg/ml), lycopene can lose its antioxidative properties and potentially generate oxidative DNA damage in cultured human colon cancer cell lines [12]. 

In our current study, lycopene extract demonstrated antimicrobial effects on various oral microorganisms, particularly *S. aureus* and *C. albicans*. This finding aligns with previous studies that have shown the impact of tomatoes on fungal organisms such as *Aspergillus niger* and *C. albicans* [13]. Although the antimicrobial properties of lycopene have been reported before, the exact underlying mechanism remains unclear. Al-Oqaili et al. have suggested that the antimicrobial activity of tomato extract is attributed to the presence of active constituents within the extract that acts against different bacteria [14]. Additionally, Lee et al. have proposed that lycopene functions as a bacterial agent by inducing ROS-mediated DNA damage, particularly involving hydroxyl radicals [15].

Vitamin E refers to a group of lipophilic antioxidants known as tocopherols. Numerous studies have highlighted their advantageous effects in respiratory tract infections, chlamydiosis, and bacterial infections caused by *Escherichia coli* and *Helicobacter pylori* [16]. Naguib et al. demonstrated that vitamin E can enhance the bactericidal effects of antibiotics by interfering with lipocalin binding [17]. In our current study, we found that vitamin E exhibited the strongest antimicrobial effect against *C. albicans* compared to other oral pathogens. Previous studies have shown positive results with vitamin E concentrations ranging from 50 to 400 IU/ml against *S. aureus* and *Staphylococcus epidermidis* [18]. However, in our study, the zone of inhibition for *S. aureus* was lower compared to *C. albicans*. This discrepancy may be attributed to differences in the concentration of vitamin E used in both studies. Despite extensive literature search, the exact molecular mechanisms underlying the antimicrobial properties remain largely unknown.

In our current study, the combined use of lycopene and vitamin E demonstrated superior antimicrobial effects compared to their individual use at the lowest concentration. The combination at the lowest concentration (25 μl) exhibited the maximum action against *C. albicans* and *S. mutans* showing the highest susceptibility. Interestingly, our literature search did not uncover any prior studies evaluating the antimicrobial effects of the lycopene and vitamin E combination. This highlights the novelty of our findings and the need for further research in this area. Further, the incorporation of naturally occurring compounds in combination therapies has revolutionized the efficacy and bioavailability of these products, as well as reducing the need for higher doses and prolonged treatment periods.

Limitations

The antimicrobial activity was assessed in vitro using agar-based assays, which may not fully represent the complex interactions that occur in a living organism. Further studies, including in vivo experiments and molecular studies, are necessary to validate the findings. While the study demonstrates promising antimicrobial effects, the clinical relevance and potential applications of lycopene and vitamin E combination in treating oral infections are not fully explored. Clinical trials are needed to assess the effectiveness and safety of this combination in a clinical setting. Addressing these limitations in future research can enhance the understanding and application of these combinations as antimicrobial agents.

## Conclusions

Our study revealed that lycopene and vitamin E combination exhibited remarkable antimicrobial activity, with *Candida albicans* being particularly susceptible. These findings suggest that lycopene could serve as a valuable adjunct to existing antimicrobial therapies. Furthermore, the combination of lycopene and vitamin E extracts, when utilized alongside other medications, holds the potential for formulating topical or systemic treatments for oral diseases. These formulations could provide clinicians with additional tools for managing various oral conditions.
